# Preparation and Characterization of Melamine Aniline Formaldehyde-Organo Clay Nanocomposite Foams (MAFOCF) as a Novel Thermal Insulation Material

**DOI:** 10.3390/polym16243578

**Published:** 2024-12-21

**Authors:** Ahmet Gürses, Elif Şahin

**Affiliations:** 1 Department of Chemistry Education, K.K. Education Faculty, Ataturk University, Erzurum 25240, Turkey; 2 Nano Science and Nano Engineering Department, Ataturk University, Erzurum 25240, Turkey; sahine@atauni.edu.tr

**Keywords:** melamine formaldehyde, aniline formaldehyde, melamine aniline formaldehyde, organoclay, polymer nanocomposite, foam, thermal insulation, compressive strength

## Abstract

The main purpose of this study is to prepare a melamine aniline formaldehyde foam, an MAF copolymer, with lower water sensitivity and non-flammability properties obtained by the condensation reaction of melamine, aniline, and formaldehyde. In addition, the preparation of MAFF composites with organoclay reinforcement was determined as a secondary target in order to obtain better mechanical strength, heat, and sound insulation properties. For the synthesis of foams, the microwave irradiation technique, which offers advantages such as faster reactions, high yields and purities, and reduced curing times, was used together with the heating technique and the effect of organoclay content on the structural and textural properties of foams and both heat insulation and mechanical stability was investigated. Virgin melamine formaldehyde foam, MFF, melamine aniline formaldehyde foam, MAFFF, and melamine aniline formaldehyde–organoclay nanocomposite foams prepared with various organoclay contents, MAFOCFs, were characterized by HRTEM, FTIR, SEM, and XRD techniques. From spectroscopic and microscopic analyses, it was observed that organoclay flakes could be exfoliated without much change in the resin matrix with increasing clay content. In addition, it was determined that aniline formaldehyde, which is thought to enter the main polymer network as a bridge, caused textural changes in the polymeric matrix, and organoclay reinforcement also affected these changes. Although the highest compressive strength was obtained in MAFOCF5 foam with high organoclay content (0.40 MPa), it was determined that the compressive strengths in the nanocomposites were generally quite high despite their low bulk densities. In the prepared nanocomposite with 0.30% organoclay content (MAFOCF2), 0.33 MPa compressive strength and 0.051 thermal conductivity coefficient were measured. For virgin polymers and composites, bulk density, thermal conductivity, and compressive strength values were determined in the order of magnitude as MFF > MAFOCF1 > MAFOCF5 > MAFOCF6 > MAFF > MAFOCF3 > MAFOCF2 > MAFOCF4; MFF > MAFF > MAFOCF6 > MAFOCF5 > MAFOCF1 > MAFOCF4 > MAFOCF3 > MAFOCF2 and MAFOCF5 > MAFOCF4 > MAFOCF2 > MAFF > MAFOCF6 > MFF > MAFOCF1 > MAFOCF3. As a result, both compressive strength and thermal conductivity values indicate that nanocomposite foam with 0.20 wt% organoclay content can be a promising new insulation material.

## 1. Introduction

Properties such as crack resistance, ductility, and thermal stability of thermoset resins, which exhibit hardness, brittleness, and low toughness due to excessive crosslink density, can be improved by adding various reinforcements such as nanofillers and -fibers and using the copolymerization process [1,2,3,4]. However, nanomaterials and -fibers in particular can easily agglomerate due to their high specific surface area fibrous structures, and preventing their agglomeration is extremely important for their effective adhesive interactions with polymer molecules [4,5,6,7,8]. Melamine formaldehyde (MF) resins, which have excellent thermal stability and high aging resistance, have advantages such as low cost and versatile usability as leather tanning and coating material, as well as high flame retardancy, advanced sound and heat insulation, low density, and high void volume [9,10]. They have higher application potential compared to traditional organic foams in fields such as construction, transportation, and aviation. Melamine formaldehyde prepolymers need to be cured by heating to gradually connect the existing oligomers with methylene and ether bridges to form a three-dimensional network structure. On the other hand, reinforcements such as nanoparticles and -fibers and different polymers have the ability to form a steric barrier against the proximity of triazine rings in the melamine formaldehyde polymer network. In particular, nanoparticles added at very low rates and uniformly dispersed in the polymeric matrix can absorb some of the impact energy generated by an external force and thus provide increased strength. In order to provide superior properties such as high strength, high thermal insulation performance, good wettability, high thermal stability, toughness, flame retardancy to melamine formaldehyde resins, clay minerals, various oxides, carbon nanotubes, graphite nanofillers, and cellulose nanofibers, oxide nanoparticles such as SiO_2_, ZnO, Al_2_O_3_, and TiO_2_ and metals such as Fe and Ni are widely used as reinforcements. In particular, organo-montmorillonite produced from montmorillonite clay, which has the advantages of low cost, abundance and prevalence, high aspect ratio, and excellent particle–surface interaction potential, can significantly improve the thermal and chemical stability and processability of thermosets [7,11,12,13,14,15,16,17,18,19,20]. On the other hand, the addition of flexible short-chain polymers such as starch, carbon fiber, polyvinyl alcohol, and polyurethane to melamine formaldehyde resin, which is highly brittle due to its molecular structure containing a large number of triazine rings and short flexible carbon chains, is also a common modification process. It has been observed that modified rigid closed-cell resins, whose flexibility is increased by increasing the flexible chain length between triazine rings, can exhibit better compressive strength, flexural strength, cellular structure integrity, thermal insulation performance, and flame resistance compared to unmodified melamine formaldehyde foams. Modifiers, which generally contain various functional groups that can react with hydroxy methyl groups or amino groups, can create less crosslink density than methylene linkage by forming links between triazine rings and thus increase the flexibility of the resin network [21,22]. There are many studies on various copolymers synthesized by condensing a mixture of phenol or various amines and formaldehyde [23,24]. These modified and copolymer resins have practical applications in many fields such as electronics, insulation materials, protective adhesives, and aerospace industries due to their high thermal stability, heat and chemical resistance, and electrical insulation properties. Melamine and aniline formaldehyde resins are well-known thermoset resins, and the most obvious advantage of aniline formaldehyde over melamine formaldehyde is its high resistance to water. Therefore, the presented study aims to characterize the surface morphological, structural, and textural properties of a new foam, both a terpolymer foam synthesized from melamine, formaldehyde, and aniline (MAFF) and its organoclay nanocomposite foams (MAFOCFs) with different organoclay ratios, and to investigate their thermal insulation behaviors and mechanical stability.

## 2. Materials and Methods

### 2.1. Materials

Raw montmorillonite (MMT), supplied from Karakayalar A.Ş. in Çankırı province, Turkey, with a specific surface area of 64.2 m^2^/g and X-ray Fluorescence (XRF) composition given in Table 1, was used for the synthesis of organo-montmorillonite (OMMT) to be used in the preparation of nanocomposites [25].

Melamine, aniline (99.5% wt.), formaldehyde (37% wt.), NaOH, acetic acid, Tween 80, a nonionic surfactant (polyoxyethylene (20) sorbitan monooleate), and analytical-grade glycerin (all supplied by Merck KGaA, Darmstadt, Germany) were used to prepare melamine formaldehyde (MFF), melamine aniline formaldehyde (MAFF) and composite foams (MAFOCFs). In addition, gasoline, a mixture of isooctane, butane, and 3-ethyltoluene used for foaming, was also supplied by a local gas station.

### 2.2. Method

#### 2.2.1. Organoclay Synthesis

Montmorillonite (MMT), a cationic surfactant, cetyltrimethylammonium bromide, and CTAB (Merck Co.) suspensions, a hydrocarbon material that is a product of petroleum refining and some of the properties of which are given in Table 2, were used to prepare organoclay using the solution intercalation method [25]. The procedure applied for the synthesis of organo-montmorillonite (OMMT) is the same as in our previous work.

#### 2.2.2. Preparation of Melamine Formaldehyde Prepolymer (MF), Melamine Aniline Formaldehyde Prepolymer (MAF), and MAF–Organoclay Nanocomposites

A mixture of melamine, aniline (99.5 wt%), and formaldehyde (37 wt%) (mass ratio: 0.67:0.02:0.31) was placed in a flat-bottomed flask equipped with a thermometer and cooling equipment and heated with stirring at approximately 60 °C until a clear solution was obtained. Then, the pH of the mixture was adjusted to 8.5 with a concentrated NaOH solution and refluxed at approximately 95 °C for 1 h. After neutralization using concentrated acetic acid, the prepolymer containing 1.0 wt% glycerin, 1.0 wt% Tween 80, and 6.0 wt% gasoline was mechanically stirred for homogenization. Virgin melamine formaldehyde resin (MF) was also prepared by the same procedure using melamine and formaldehyde (37 wt%) in a mass ratio of 0.68:0.32. In the preparation of resin–organoclay nanocomposites by in situ synthesis, organoclay was added to the flask containing other components at weight ratios of 0.10, 0.15, 0.20, 0.30, 0.40, and 0.45%. For the foaming process, mixtures with and without organoclay reinforcement were exposed to microwave radiation in a microwave oven for 2 min in a suitable container. Finally, the mixtures were placed in a modular aluminum mold of 10.0 cm × 10.0 cm × 1.0 cm and heated in an oven at 140 °C for 1 h. The samples were prepared in duplicate. The compositions and codes of virgin MFF and MAFF and MAFOCFs are presented in Table 3.

#### 2.2.3. Spectroscopic and Microscopic Analyses and Measurements of Compressive Strength and Thermal Conductivity

Structural, crystallographic, and textural characterization of virgin MFF foam, virgin MAF foam, and MAF–organoclay nanocomposite foams were performed using spectroscopic techniques such as XRD and FTIR and microscopic techniques such as HRTEM and SEM.

XRD diffractograms for the prepared samples were obtained using a PANalytical Empyrean X-ray diffractometer with Cu Ka (1540 Å) radiation operating at 5 kV and 40 mA for 2 h in the 9–90° range and a scan rate of 4/min (Malvern Panalytical Ltd., Malvern, UK).

FTIR spectra were obtained using a Vertex 70 V FTIR spectrometer with a mid-IR ceramic source in the range of 4000 to 400 cm^−1^, an average of 100 scans, and a resolution of 1 cm^−1^ (Bruker Optics Inc., Billerica, MA, USA).

HRTEM images of the samples were taken using a HITACHI HT7700 high-resolution transmission electron microscope (LaB6 filament) operating at 120.0 kV (Hitachi Ltd., Tokyo, Japan).

SEM (FEI-INSPECT S50 model) at 30 kV was used to take SEM patterns of virgin MFF, virgin MAFF, and MAF–organoclay nanocomposite foams.

To measure the compressive strength of virgin MFF and MAFF and MAFOCFs, a universal testing machine (Zwick/Roell) was used according to the DIN ISO 844:2009-10 standard [17,20].

A thermal conductivity meter (Quick Thermal Conductivity Meter QTM-500, Minami-ku, Kyoto, Japan) with a probe consisting of a single heating wire and a thermocouple was used to measure the thermal conductivity coefficients of virgin MFF and MAFF and MAFOCFs. The sample thickness was 8.0 mm, and the measurements were made in duplicate.

## 3. Results and Discussion

### 3.1. Textural Structure Analysis of Melamine Formaldehyde Foam (MFF), Melamine Aniline Foam (MAFF), and Melamine Aniline Copolymer Organoclay Nanocomposite Foams (MAFOCFs1-6)

**Figure 1:**
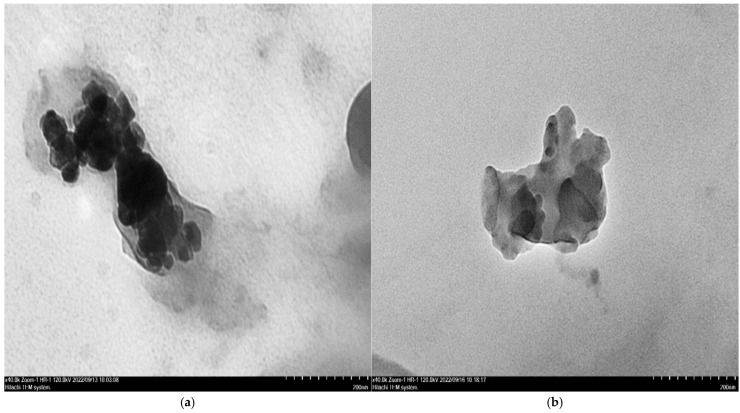

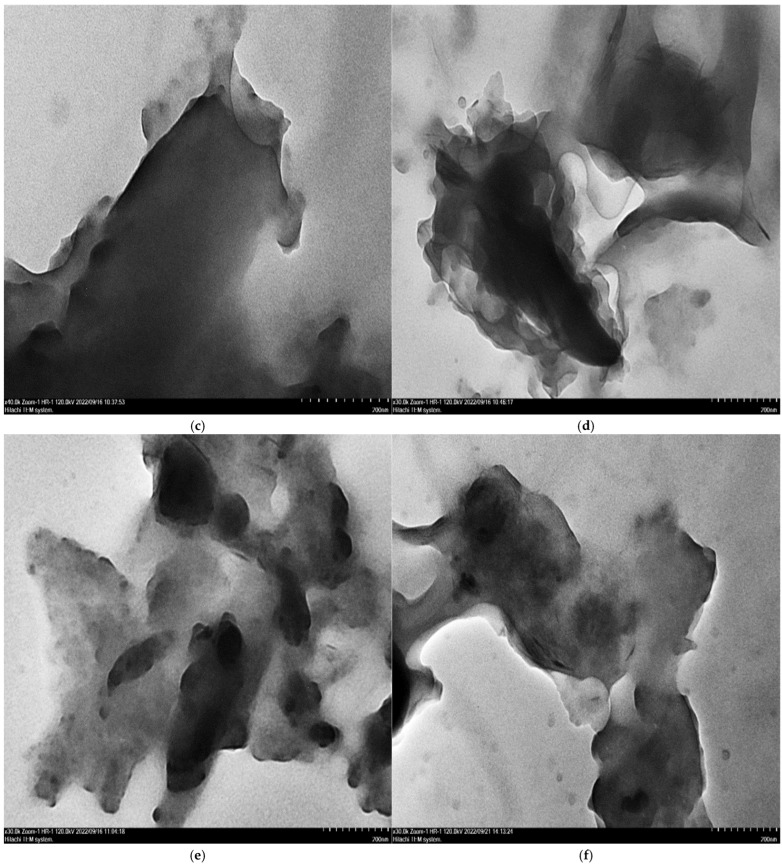

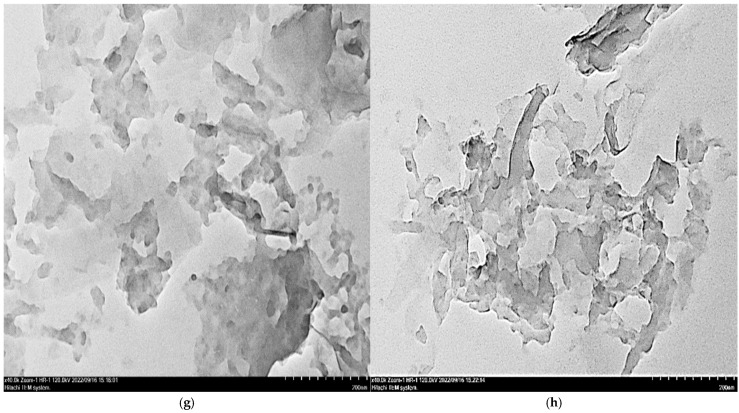
HRTEM images of virgin melamine formaldehyde foam (MFF) (**a**), virgin melamine aniline co-polymer foam (MAFF) (**b**), and melamine aniline copolymer organoclay nanocomposite foams (MAFOCFs1-6) (**c**–**h**).

For the textural characterization of melamine formaldehyde foam (MFF), melamine aniline copolymer foam (MAFF) foam, and melamine aniline copolymer organoclay nanocomposite foams (MAFOCFs1-6) prepared with various organoclay contents, HRTEM images are given in Figure 1.

From Figure 1a, it can be seen that in virgin MFF, the aggregated resin microspheres interact with each other and form a cluster-like growth in three dimensions. This was associated with the emergence of micro water vapor bubbles by the use of microwave irradiation before curing by heating. From Figure 1b, it can be seen that in virgin MAFF, a relatively planar structure emerges, reflecting the formation of aniline–formaldehyde bridges, which probably cause the triazine rings to be more distant from each other, as well as the presence of more effective particle interactions, compared to virgin MFF. The images of the composites prepared with various organoclay contents in Figure 1c–h show that increasing the organoclay content produces more pronounced textural changes compared to virgin MAFF, and the appearance of organoclay platelets, depicted as dark lines. From Figure 1d, it can be seen that despite the organoclay platelets tending to partially aggregate, they are predominantly exfoliated, resulting in a textural structure with different topographic forms. In Figure 1e,f, it is seen that, despite the higher organoclay contents, the organoclay platelets are almost completely exfoliated, which leads to the emergence of a more regular structure and a more planar appearance of the topographic forms. This situation is explained by the fact that aniline formaldehyde groups are included in the network in the form of bridging in the formation of the melamine formaldehyde polymeric network, thus causing the planar structure to dominate, in contrast to the possibility of increasing bending capabilities. In the images of the composites with higher organoclay contents (MAFOCF5 and MAFOCF6), a textural structure is observed in which the organoclay platelets are more homogeneously dispersed and more rectangular pyramidal topographic arrangements are formed in the resin.

### 3.2. Surface Morphological Analysis of Melamine Formaldehyde Foam (MFF), Melamine Aniline Copolymer Foam (MAFF), and Melamine Aniline Copolymer Organoclay Nanocomposite Foams (MAFOCFs1-6)

**Figure 2:**
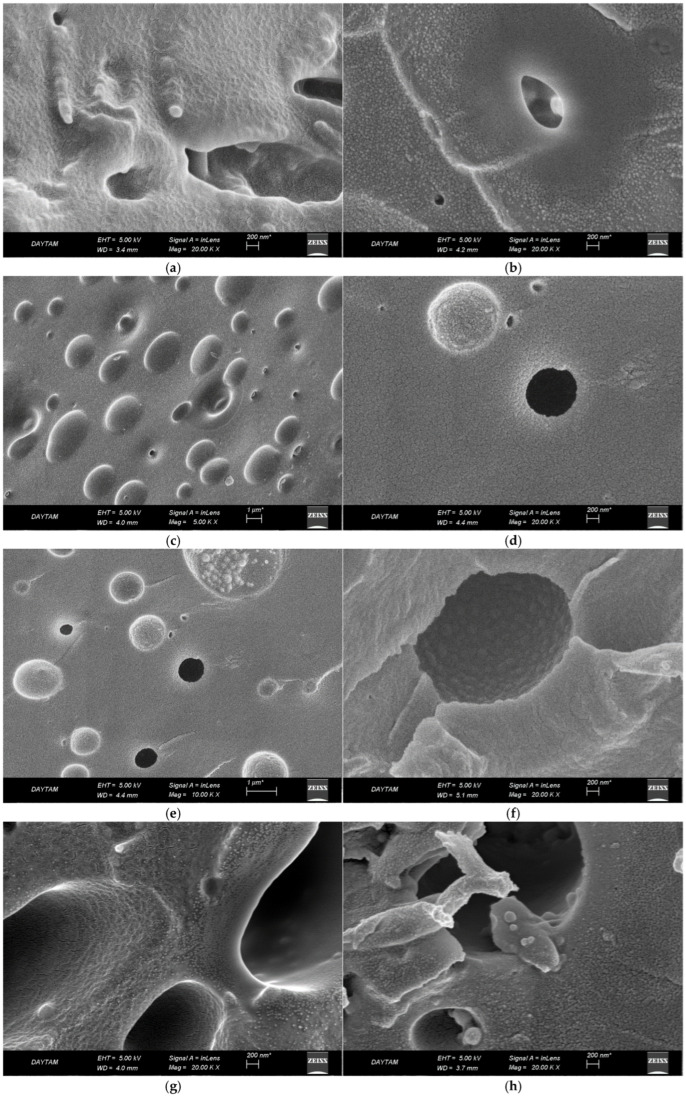
SEM patterns of virgin melamine formaldehyde foam (MFF) (**a**), virgin melamine aniline copolymer foam (MAFF) (**b**), and melamine aniline copolymer organoclay nanocomposite foams (MAFOCFs1-6) (**c**–**h**).

The SEM patterns taken for the surface morphological characterization of melamine formaldehyde foam (MFF), melamine aniline copolymer foam (MAFF) foam, and melamine aniline copolymer organoclay nanocomposite foams (MAFOCFs1-6) prepared with various organoclay contents are presented in Figure 2. The pattern of MFF in Figure 2a reflects a surface morphology indicating the resin microsphere clusters, grown in three dimensions and interacting with each other [17,20]. It can be seen from Figure 2b that MAFF exhibits a more regular morphology compared to MFF, and a network structure is formed in MAFF indicating the coherent association of relatively smaller resin microspheres. The surface morphology of MAFOCF with low organoclay content shows the formation of elliptical and probably tubular cavities and a surface morphology reflecting a regular network structure formed by the aggregation of very small resin microspheres with higher compliance, and significant changes compared to that of virgin MAFF (Figure 2c). The surface morphologies of MAFOCFs given in Figure 2d–g show that the aggregation of very small resin microspheres with higher compliance leads to the formation of a more holistic structure and the elliptical cavities gradually become circular. This shows that the organoclay platelets dispersed in the polymeric matrix are significantly effective in both the porosity development and the topographic orientations of the polymer skeleton. From Figure 2h, it can be seen that in the composite coded MAFOCF6, morphological formations in the form of defects with different network structures, probably resulting from the heterogeneous distribution of organoclay platelets, occur [26].

### 3.3. Structural Analysis of Melamine Formaldehyde Foam (MFF), Melamine Aniline Foam (MAFF), and Melamine Aniline Copolymer Organoclay Nanocomposite Foams (MAFOCFs1-6)

**Figure 3:**
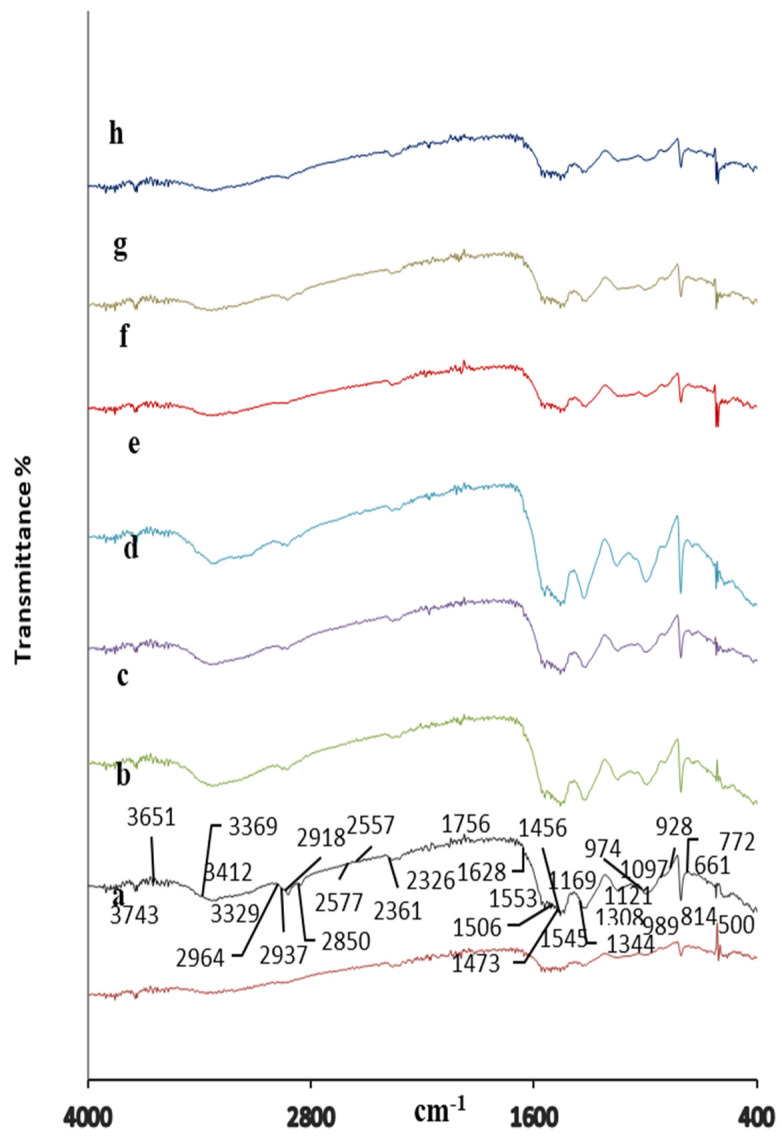
FTIR analysis of virgin melamine formaldehyde foam (MFF) (**a**), virgin melamine aniline formaldehyde foam (MAFF) (**b**), and melamine aniline formaldehyde-organoclay nanocomposite foams (MAFOCFs1-6) (**c**–**h**).

FTIR spectra of virgin MFF and MAFF and resin–organoclay nanocomposite foams (MAFOCF1, MAFOCF2, MAFOCF3, MAFOCF4, MAFOCF5, and MAFOCF6) are given in Figure 3.

The peaks at 3329 cm^−1^, 989 cm^−1^, 1545 cm^−1^, and 1456 cm^−1^ in the FTIR spectrum of MFF, MAFF, and also MAFOCF correspond to the stretching vibrations of N-H, O-H, C-O-C, and C-N bonds, respectively [27]. From Figure 3, it can be seen that in the spectrum of the MAF–organoclay nanocomposite foams (MAFOCFs), there was a strong peak at 3329 cm^−1^ originating from CTAB as well as the peaks at 2326 cm^−1^, 2361 cm^−1^, and 2937 cm^−1^, which correspond to C–H anti-stretching, C–H stretching, and amine group stretching vibrations. The peaks observed between 999 cm^−1^ and 500 cm^−1^ correspond to the bonding vibrations of the main functional groups Si-O and Al-OH. It is observed that the intensity of these peaks increases with increasing organoclay content. The relatively strong, broad peak at 3329 cm^−1^ and the peak at 3743 cm^−1^ indicate the presence of hydroxyl and N–H bonds, and the peak intensities increased with increasing organoclay content [17,20].

On the other hand, the relatively low intensities of these peaks can be attributed to the intense interactions between the clay layers and CTA^+^ ions bound to long-chain hydrocarbons. The peaks at 1545 cm^−1^ and 814 cm^−1^ are characteristic of the triazine ring of melamine. The fact that the spectra of MAFF and MAFOCFs are almost similar may imply that the addition of organoclay does not affect the functional groups on the polymer surface and that they are sterically effective. The peaks at 1558 cm^−1^ and 3400 cm^−1^ observed in the spectra of MAFOCFs correspond to cyclic aromatic C=C vibrations and N-H stretching, indicating the incorporation of aniline into the polymer backbone [28,29]. The peaks at 500 cm^−1^ and 444 cm^−1^ can be associated with Si–O–Al (octahedral Al) and Si–O–Si bending vibrations originating from organoclay. The peaks observed in the range of 2850–2964 cm^−1^ correspond to the antisymmetric and symmetric stretching of CH_2_ groups of the CTA^+^ ions on the organoclay surface [30].

### 3.4. XRD Analysis of Melamine Formaldehyde Foam (MFF), Melamine Aniline Copolymer Foam (MAFF), and Melamine Aniline Copolymer Organoclay Nanocomposite Foams (MAFOCFs1-6)

**Figure 4:**
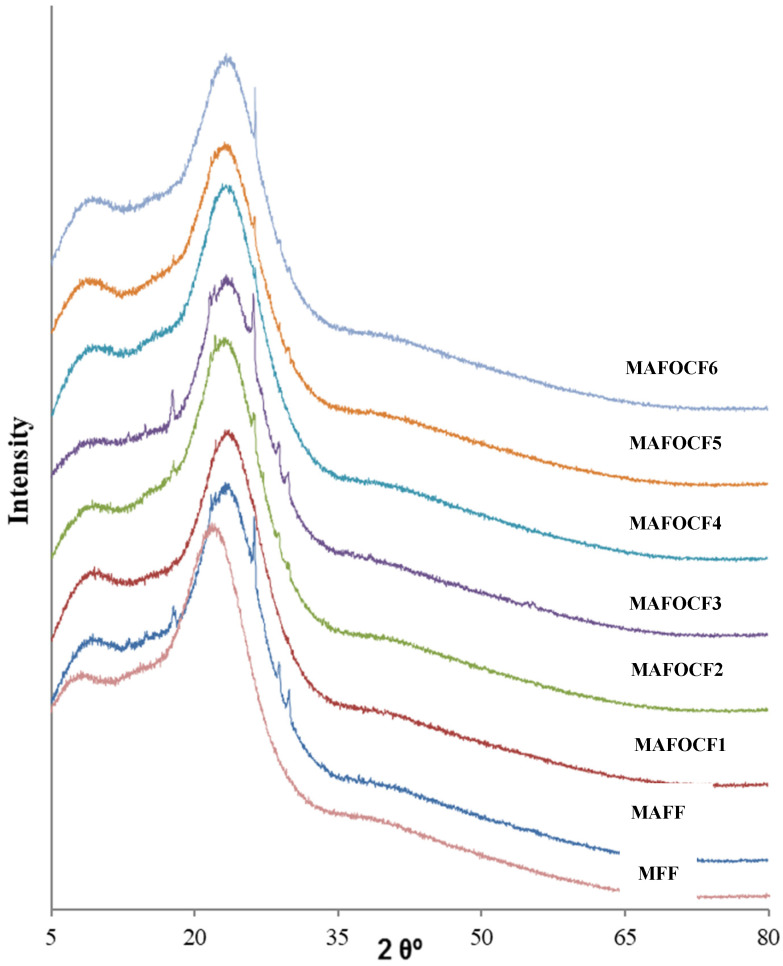
XRD diffractograms of virgin melamine formaldehyde foam (MF), virgin melamine aniline formaldehyde foam (MAFF), and melamine aniline formaldehyde–organoclay nanocomposite foams (MAFOCFs1-6).

The XRD diffractograms of melamine formaldehyde foam (MFF), melamine aniline copolymer foam (MAFF), and melamine aniline copolymer organoclay nanocomposite foams (MAFONCFs1-6) are given in Figure 4. The two typical broad peaks at 8.0° and 21.9° in the XRD pattern of MFF indicate the amorphous structure of the resin [17,20]. The peaks at 18.4°, 21.1°, 26.3°, 28.0°, and 29.3° in the diffractogram of virgin MAFF indicate the incorporation of aniline and formaldehyde into the polymer network, probably in the form of bridging [31,32]. On the other hand, it can be seen in Figure 4 that the characteristic smectite peak observed at approximately 10° in organoclay-reinforced MAFOCsF1-6 partially overlaps with the smaller peak belonging to MF and this peak also shifts to the left in composites with low organoclay content. The relative shift in the characteristic smectite peak to the left indicates that the organoclay platelets in the polymer matrix are exfoliated. In addition, in the diffractograms of the resin–organoclay composite foams, the peaks that emerged with the addition of aniline formaldehyde to the amorphous MF polymer network are shown to have decreased in intensity, or some of them disappeared with organoclay reinforcement, which reveals the effect and decisive role of organoclay platelets in the change in crystallographic structure.

### 3.5. Analysis of Thermal Insulation Characteristics of Virgin Melamine Formaldehyde Foam (MF), Virgin Melamine Aniline Formaldehyde Foam (MAFF), and Melamine Aniline Formaldehyde–Organoclay Nanocomposite Foams (MAFOCFs1-6)

Thermal conductivity is an important property used to evaluate the thermal performance of a material under steady-state conditions. The thermal insulation performances of the prepared foams were evaluated by measuring their thermal conductivity coefficients [33,34,35,36]. Table 4 shows the bulk density and thermal conductivity values of virgin MFF and MAFF and resin–organoclay composite foams with different organoclay contents, such as MAFOCFs1-6. From this table, it can be seen that the lowest conductivity coefficients are exhibited by composite foams with high organoclay content, such as MAFOCF2, MAFOCF3, and MAFOCF4. This behavior, which may be related to the formation of a very regular and holistic textural structure, indicates the decisive effect of the exfoliated organoclay platelets on the formation of the polymeric network structure reflecting the void textural organization. It can be said that, especially in these composite foams, the transformation of the textural network formed by the aggregation of microspheres from sphericity to a relatively planar or layered structure may have led to the emergence of a more effective barrier system in terms of heat conduction with a more tortuous arrangement (see Figure 1c–e). The calculated bulk density values of 0.16, 0.17, and 0.15 g/cm^3^ for these composite foams are significantly lower than those of virgin MFFs and MAFFs, respectively, confirming the significant and void-friendly textural changes and the formation of regular voids by the incorporation of organoclay platelets into the matrix. On the other hand, MAFOCF5 and -6-coded foams with higher organoclay content exhibited relatively higher thermal conductivity values compared to other composite foams, with lower thermal conductivity values compared to virgin MFF foam, which was attributed to possible textural changes leading to partial disruption of the regular void structure due to the increase in organoclay content. In particular, the better thermal insulation behavior of virgin MAFF compared to virgin MFF indicates the effect of a more uniformly packed microsphere arrangement and more elastic network structure including aniline–formaldehyde bridges on the formation of the void structure. The approximately 45% increase in bulk density observed in composites with higher organoclay content may indicate a significant disruption in the structural arrangement producing voids due to textural change.

### 3.6. Analysis of Compressive Strengths of Virgin Melamine Formaldehyde Foam (MF), Virgin Melamine Aniline Formaldehyde Foam (MAFF), and Melamine Aniline Formaldehyde–Organoclay Nanocomposite Foams (MAFOCFs1-6)

The compressive strength of materials is a critical design property measured under compressive loads and is also used to characterize the structure of composite materials [37,38]. There are differences in compressive fracture behavior between brittle materials, which are prone to splitting and sudden fracture, and ductile materials, which are prone to flow without fracture. Thermoset resins and their composites are brittle materials. In many materials, mechanical stability depends on porosity, and microcracks can grow until their dimensions are the same as the pores. Even at low porosity and hardness, microcracks can develop between weak interfaces under load, and these interfaces can propagate and form new cracks [39,40,41]. Table 5 shows the compressive strengths of virgin MFF and MAFF and composite foams prepared with various organoclay ratios, MAFOCFs1-6, obtained from measurements made on two replicates and 8.0 mm thick samples, together with standard deviation values. As can be seen from Table 5, the compressive strength of MAF foam is higher compared to MF foam. This means that the increase in elasticity caused by the inclusion of aniline–formaldehyde bridges in the melamine formaldehyde polymer network positively affects its mechanical stability, thus making the MAF foam more ductile. In addition, it can be seen from the same table that the foam with the highest organoclay content, MAFOCF6, has significantly lower compressive strength compared to other composite foams with both relatively high densities and high thermal conductivity coefficients. It is known that microcracks are the source of mechanical weakness in brittle materials and that slippage along the cracks causes tensile forces along the cracks. Microcracks, which develop for different reasons, usually occur close to the crack tips and can interact with microstructural anomalies, leading to energy accumulation. Therefore, the weakened mechanical strength of MAFOCF6 with high organoclay content can be attributed to microcracks that may have occurred due to anomalies such as planar textural changes and the formation of small polymeric fragments due to the dispersion of organoclay platelets dispersed in the resin (see Figure 2h).

## 4. Conclusions

This study aims to prepare and analyze the structural and textural properties of melamine aniline formaldehyde copolymer organoclay nanocomposite foams and to investigate both their thermal insulation and strength properties.

Melamine aniline formaldehyde foam and melamine aniline formaldehyde–organoclay nanocomposites were successfully prepared. It was determined that the addition of organoclay caused significant changes in the textural structure of the resin.

In the lowest organoclay content nanocomposite foam, MAFOCF1, exfoliation and partial aggregation of organoclay occurred, but it was determined that this nanocomposite was similar to virgin MAFF in terms of textural and morphological features.

From HRTEM and SEM images, except for MAFOCF6, a more regular structural arrangement was observed with increasing organoclay content (MAFOCF2 and -5 nanocomposites), indicating more advanced organoclay exfoliation. From the SEM patterns, it was observed that the surface morphologies of MAFF with high organoclay content reflected the formation of a holistic structure by effective and harmonious aggregation of small microspheres, and the elliptical cavities gradually became circular.

From the XRD spectra of virgin MAFF and MAFOCFs, it was observed that in nanocomposites, the smaller of the two peaks of MAFF partially overlapped with the characteristic smectite peak, and the smectite peak was partially shifted to the left, implying intercalated or exfoliated dispersion of organoclay platelets.

The better thermal insulation behavior of virgin MAFF compared to virgin MFF indicates the effect of a more uniformly packed microsphere arrangement and more elastic network structure including aniline–formaldehyde bridges on the formation of void structure. The thermal insulation values gradually decreased with the increase in organoclay content in resin–organoclay nanocomposites.

The mechanical stability of virgin MAFF and resin–organoclay nanocomposites was relatively higher than that of virgin MFF, indicating that the increase in elasticity caused by the incorporation of aniline–formaldehyde bridges into the polymer network had a positive effect in terms of ductility and mechanical stability. Moreover, MAFOCF2-5-coded resin–organoclay nanocomposites exhibited significantly higher compressive strength compared to virgin resin foams and other composite foams despite having low density and low thermal conductivity coefficients.

## Figures and Tables

**Table 1:** X-ray Fluorescence (XRF) chemical compositions of montmorillonite (MMT).

*Component (%)*									
SiO_2_	Al_2_O_3_	Fe_2_O_3_	MgO	CaO	Na_2_O	K_2_O	TiO_2_	SO_3_	Other
59.32	17.19	5.95	3.63	2.21	1.68	0.97	0.74	0.51	7.81

**Table 2:** Some characteristics of the hydrocarbon material [25].

Density (15 °C), kg/m^3^	Calorific Value MJ/kg	Flash Point °C	Water by Distillation, wt. %	C	H	N	S	Ash
990.70	42.74	105.80	0.10	83.40	11.90	0.80	1.50	0.03

**Table 3:** Codes and compositions of virgin MFF and MAFF and MAFOCF1-6 foams.

Sample No.	Sample Code	Organoclay wt. %
1	Melamine formaldehyde foam MFF	-
2	Melanin aniline formaldehyde foam MAFF	-
3	Melamine aniline formaldehyde–organoclay nanocomposite foam1 MAFOCF1	0.10
4	Melamine aniline formaldehyde–organoclay nanocomposite foam2 MAFOCF2	0.15
5	Melamine aniline formaldehyde–organoclay nanocomposite foam3 MAFOCF3	0.20
6	Melamine aniline formaldehyde–organoclay nanocomposite foam4 MAFOCF4	0.30
7	Melamine aniline formaldehyde–organoclay nanocomposite foam5 MAFOCF5	0.40
8	Melamine aniline formaldehyde–organoclay nanocomposite foam6 MAFOCF6	0.45

**Table 4:** Bulk densities and thermal conductivities of virgin MFF and MAFF and composites, such as MAFOCFs1-6, prepared with various organoclay ratios.

Codes of Specimen	Bulk Density g/cm^3^	Thermal Conductivity Coefficient λ, W/(m K)	Standard Deviation
MFF	0.26	0.083	0.001
MAFF	0.19	0.069	0.011
MAFOCF1	0.23	0.063	0.008
MAFOCF2	0.16	0.051	0.010
MAFOCF3	0.17	0.052	0.007
MAFOCF4	0.15	0.053	0.008
MAFOCF5	0.23	0.065	0.004
MAFOCF6	0.22	0.076	0.003

**Table 5:** Compressive strengths of MAFOCF1-6 composites with virgin MFF and MAFF with various organoclay ratios.

Codes of Specimen	Compressive Strength, MPa	Standard Deviation
MFF	0.27	0.020
MAFF	0.30	0.011
MAFOCF1	0.24	0.080
MAFOCF2	0.33	0.010
MAFOCF3	0.19	0.090
MAFOCF4	0.34	0.010
MAFOCF5	0.40	0.090
MAFOCF6	0.29	0.003

## Data Availability

The original contributions presented in this study are included in the article. Further inquiries can be directed to the corresponding author.
